# Inhibition of STAT3 signaling targets both tumor-initiating and differentiated cell populations in prostate cancer

**DOI:** 10.18632/oncotarget.2314

**Published:** 2014-08-06

**Authors:** Zhiqiang Han, Xiaoli Wang, Liang Ma, Lijuan Chen, Min Xiao, Liang Huang, Yang Cao, Jian Bai, Ding Ma, Jianfeng Zhou, Zhenya Hong

**Affiliations:** ^1^ Cancer Biology Research Center, Tongji Hospital, Tongji Medical College, Huazhong University of Science and Technology, Wuhan, Hubei, China; ^2^ Department of Urology, First Affiliated Hospital of Guangxi Medical University, Nanning, Guangxi, China; ^3^ Department of Obstetrics and Gynecology, Union Hospital, Tongji Medical College, Huazhong University of Science and Technology, Wuhan, Hubei, China; ^4^ Department of Hematology, Tongji Hospital, Tongji Medical College, Huazhong University of Science and Technology, Wuhan, Hubei, China

**Keywords:** STAT3, prostate cancer, tumor-initiating cell, ALDH, Stattic

## Abstract

Despite of tremendous research efforts to profile prostate cancer, the genetic alterations and biological processes that correlate with disease progression remain partially elusive. In this study we show that the STAT3 small molecule inhibitor Stattic caused S-phase accumulation at low-dose levels and led to massive apoptosis at a relatively high-dose level in prostate cancer cells. STAT3 knockdown led to the disruption of the microvascular niche which tumor-initiating cells (TICs) and non-tumor initiating cells (non-TICs)depend on. Primary human prostate cancer cells and prostate cancer cell line contained high aldehyde dehydrogenase activity (ALDH^high^) subpopulations with stem cell-like characteristics, which expressed higher levels of the active phosphorylated form of STAT3 (pSTAT3) than that of non-ALDH^high^ subpopulations. Stattic could singnificantly decreas the population of ALDH^high^ prostate cancer cells even at low-dose levels. IL-6 can convert non-ALDH^high^ cells to ALDH^high^ cells in prostate cancer cell line as well as from cells derived from human prostate tumors, the conversion mediated by IL-6 was abrogated in the presence of STAT3 inhibitor or upon STAT3 knockdown. STAT3 knockdown significantly impaired the ability of prostate cancer cells to initiate development of prostate adenocarcinoma. Moreover, blockade of STAT3 signaling was significantly effective in eradicating the tumor-initiating and bulk tumor cancer cell populations in both prostate cancer cell-line xenograft model and patient-derived tumor xenograft (PDTX) models. This data suggests that targeting both *tumor initiating and differentiated cell populations* by STAT3 inhibition is predicted to have greater efficacy for prostate cancer treatment.

## INTRODUCTION

Prostate cancer is the most frequently diagnosed cancer and the second most common cause of cancer related deaths in men worldwide [1]. Although initially treatable, prostate cancer can recur in an androgen-insensitive or hormone-refractory form that is not responsive to current available therapies. The mortality rate associated with recurrent prostate cancer is high. Although significant progress has been made in understanding the molecular mechanisms of prostate cancer development, the specific molecular regulatory pathways involved in prostate cancer progression remain elusive. Search for effective therapies for the management of this disease has become a priority for researchers.

Tumor-initiating cells (TICs) also referred to as cancer stem cells (CSCs), have been implicated in tumor formation, progression, and therapy-resistance in multiple solid-organ cancers [2]. However, the development of prostate TICs is not well understood and identification of signaling pathways that regulate phenotypic and tumorigenic potential of TICs might provide new insights for drug development to prevent tumor drug resistance and relapse in prostate cancer [3]. ALDH is an intracellular enzyme involved in retinoic acid metabolism and its activity has been shown to enrich for normal or malignant stem cell populations in multiple organ systems [4-8]. The high ALDH expression in prostate cancer has been shown to be positively correlated with the Gleason score and inversely associated with overall survival of prostate cancer patients [7]. High ALDH activity has successfully been used to identify tumor-initiating and metastasis-initiating cells in human prostate cancer [8].

Signal Transducer and Activator of Transcription 3 (STAT3) is the STAT family of transcription factors activated by many cytokines and growth factors [9, 10, 11]. In normal cells, STAT3 modulates a variety of biological functions by activating the transcription of a diverse set of genes [12, 13]. Furthermore, STAT3 plays an important role in maintaining pluripotency and self-renewing processes in embryonic stem cells [14, 15]. In these cells, activation of the STAT3 protein is a reversible, tightly controlled process that typically lasts for a limited duration [16]. Conversely, the persistent activation of the STAT3 protein is detected at high frequency in a large number of human malignancies [17, 18, 19, 20]. Aberrantly active STAT3 contributes to oncogenesis by preventing apoptosis, inducing cell proliferation and suppressing anti-tumor immune responses [16]. These make STAT3 an excellent molecular candidate for cancer therapy.

In this study, we investigated the role of activated STAT3 signaling on differentiated cancer cell and TIC development in prostate cancer. Our findings provide new mechanistic insights for prostate cancer development.

## RESULTS

### Activated STAT3 is found in prostate cancer cell line and prostate cancer tissues

**Figure 1:**
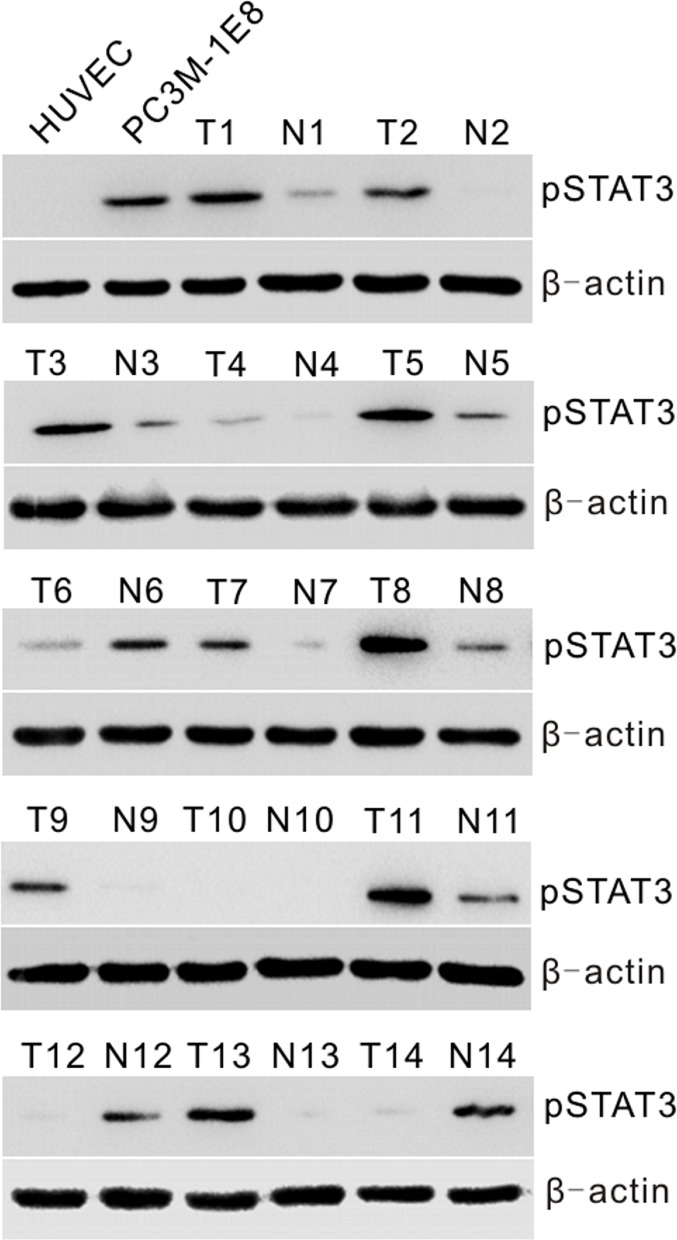
Analyses of STAT3 activation in prostate cancer cells and human primary prostate tumors Western blot analysis of pSTAT3 protein levels in HUVECs and PC3M-1E8 cells, representative snap-frozen primary prostate tumor tissues (T) and matched adjacent nontumor tissues (N).

To determine whether constitutive activation of STAT3 is associated with prostate cancer *in vitro* and *in vivo*, we first examined activated STAT3 expression by Western blotting with anti-pSTAT3 (Tyr-705) antibody in human PC3M-1E8 prostate cancer cells and a series of 14 primary prostate tumor (T) specimens with matched adjacent nontumor (N) prostate tissues. The results showed that a high pSTAT3 level was found in PC3M-1E8 cells, and higher levels of pSTAT3 were found, to different extents, in about 71% (10 of 14) of primary prostate tumor specimens as compared to matched adjacent nontumor tissues (Fig. 1).

### STAT3 small molecule inhibitor blocks STAT3 activation and suppresses STAT3-mediated gene expression in PC3M-1E8 cells

**Figure 2:**
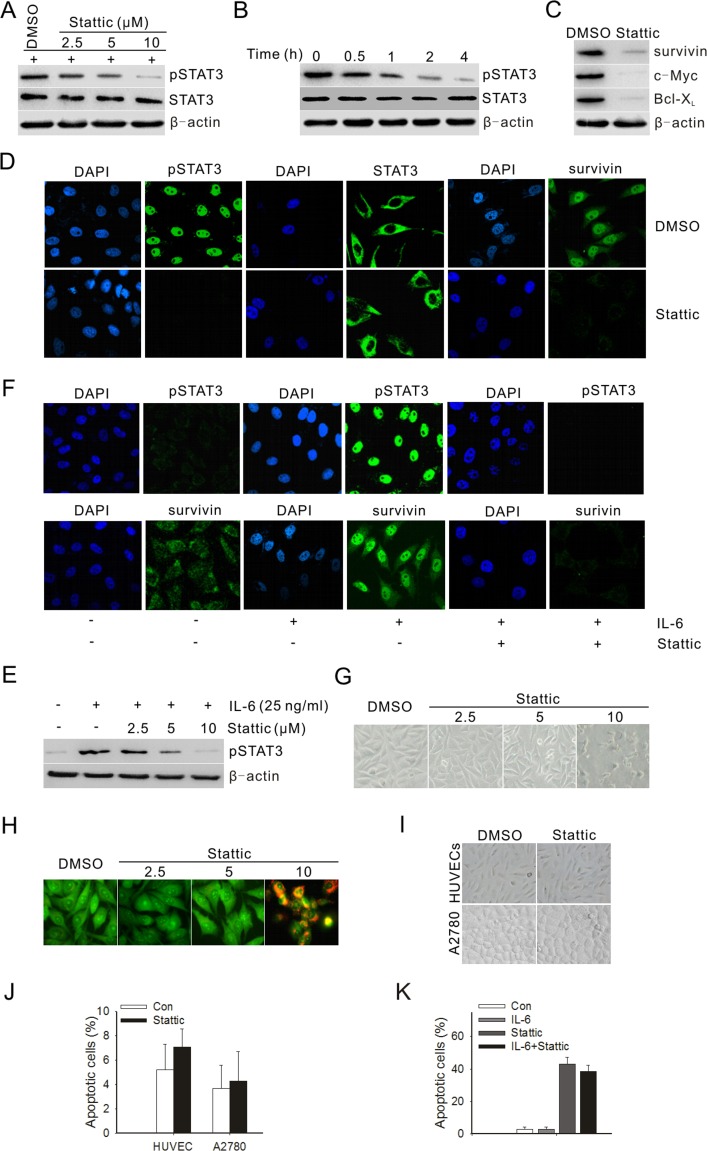
Stattic blocked constitutive and IL-6-induced activation of STAT3 and downstream targets, and suppressed both the growth and survival of prostate cancer cells (A) PC3M-1E8 cells were treated with different doses of Stattic for 4 h. (B) PC3M-1E8 cells were treated with 10 μM Stattic for different time points. (C) Western blot analysis of STAT3 downstream targets in PC3M-1E8 cells treated with 10 μM Stattic for 24 h. (D) Cellular localization of pSTAT3 and survivin (green) was observed by confocal microscopy after immunofluorescent staining of PC3M-1E8 cancer cells treated with 10 μM Stattic. (E) PC3M-1E8 cells were cultured in serum-free medium overnight, then treated with 25 ng/mL IL-6 and various amounts of Stattic for 24 hours. (F) PC3M-1E8 cells in serum-free medium were treated with 25 ng/mL IL-6 and 10 μM Stattic and examined for pSTAT3 and survivin expression by immunofluorescence (green). (G) PC3M-1E8 cells were treated with different doses of Stattic. After 48 h, morphological examinations were performed. (H) PC3M-1E8 cells were treated with different doses of Stattic. After 48 h, cells were stained with acridine orange for visualizing apoptotic cells. (I) A2780 ovarian cancer cells and HUVECs were treated with 20 μM Stattic. After 48 h, morphological examinations were performed. (J) A2780 cells and HUVECs were treated with 20 μM Stattic. After 48 h, cells apoptosis was analyzed by flow cytometry. (K) PC3M-1E8 cells were treated with 10 μM Stattic in the absence or presence of 25 ng/mL IL-6. After 48 h, cells apoptosis was analyzed by flow cytometry.

To investigate whether a STAT3 inhibitor could inhibit STAT3 activation in prostate cancer cells, PC3M-1E8 prostate cancer cells were treated with different doses of Stattic. The results showed that Stattic inhibited the STAT3 activation in a dose- and time-dependent manner (Fig. 2A and B). In contrast, the total of STAT3 showed no change (Fig. 2A and B). As was the case with STAT3 activation, Bcl-xL, survivin and c-Myc were likewise downregulated after treatment with Stattic (Fig. 2 C). Because Stattic treatment decreased the level of pSTAT3 in PC3M-1E8 cells, we then determined the effect of Stattic on nuclear translocation of pSTAT3. Immunostaining for pSTAT3 nuclear staining in PC3M-1E8 cells following 24 hours of treatment with Stattic is shown in Fig. 2D. Consistent with the immunoblotting results shown in Fig. 2A and B, the nuclear level of pSTAT3 and survivin in PC3M-1E8 cells was significantly reduced in the presence of Stattic at 10 μM concentration (Fig. 2D).

To study if IL-6 could induce STAT3 activation in PC3M-1E8 cells and be inhibited by Stattic, PC3M-1E8 cells were cultured in serum-free medium overnight and then treated with 25 ng/mL IL-6 and Stattic at indicated concentration for 24 hours. The data suggested that IL-6 induced STAT3 activation and the IL-6-induced STAT3 activation was inhibited by Stattic in a dose-dependent manner. (Fig. 2E and F).

### STAT3 activation is required for prostate cancer cells growth and survival

To explore the effect of Stattic on cell growth and apoptosis, PC3M-1E8 cells were treated with Stattic at different doses and analyzed after 48 hours. PC3M-1E8 cells showed no significant morphological changes when treated with 2.5 and 5 μM Stattic, while most of the cells became rounded and detached from the culture plates when treated with 10 μM Stattic (Fig. 2G). Figure 2H summarizes the apoptotic effects of Stattic on PC3M-1E8 cells by using the acridine orange fluorescent staining. Then, we examined the apoptosis by flow cytometric analysis and determined whether the growth inhibition was associated with specific changes in cell cycle distribution. The results displayed that PC3M-1E8 cells treated with Stattic (2.5-10 μM) showed *significant S phase accumulation* compared with the control (Table 1). 2.5μM and 5 μM Stattic did not induce significant cell apoptosis, whereas 10 μM Stattic induced 11-fold more cell apoptosis compared to the control (Table 1). Additionally, to rule out the non-specific cytotoxicity of Stattic, A2780 ovarian cancer cells and HUVECs were treated with 20 μM Stattic, which had little STAT3 phosphorylation recognized [21]. The results demonstrated that 20 μM Stattic could not lead to significant morphological changes or apoptosis in A2780 cells and HUVECs (Fig. 2I and J). Moreover, IL-6-stimulated STAT3 activation largely failed to confer resistance against Stattic-induced apoptosis (Fig. 2K).

### Blocking STAT3 activation inhibits PC3M-1E8 cells colony formation in soft agar

**Figure 3:**
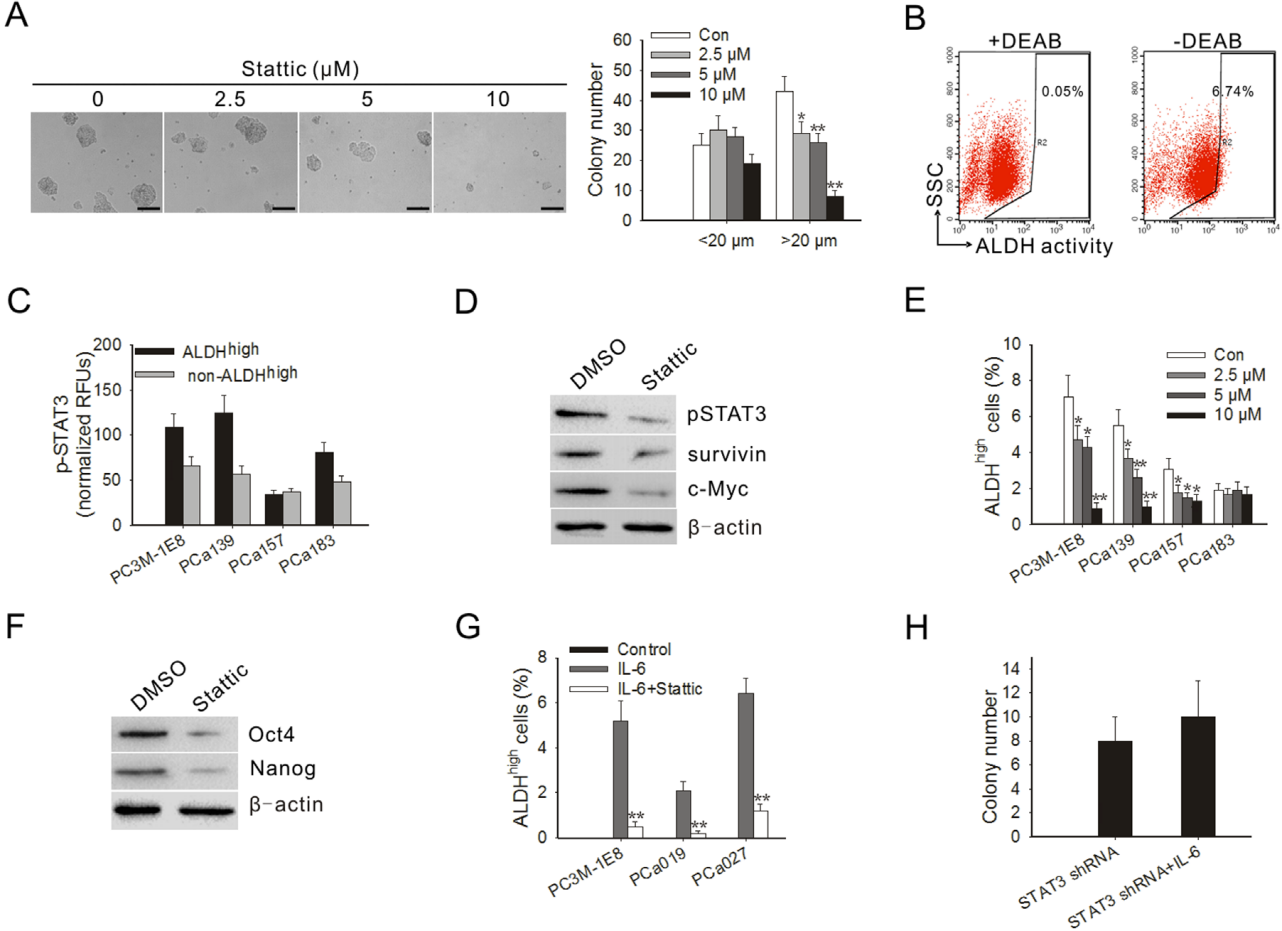
Blocking STAT3 activation inhibited anchorage-independent growth of PC3M-1E8 cells, decreased the proportion of ALDH^high^ cells and blocked the conversion of non-ALDH^high^ cells to ALDH^high^ cells mediated by IL-6 (A) Representative images of soft agar assay (left panel). Quantitation of colonies from soft agar assay of PC3M-1E8 cells treated with Stattic (right panel). (B) PC3M-1E8 cells were subjected to ALDEFLUORH assay in order to identify cells with high ALDH expression (ALDH^high^). The ALDH inhibitor DEAB was used as a negative control (left panel). The cells without inhibitor shifted to the right were considered ALDH^high^ cells (right panel). (C) ELISA for pSTAT3 in ALDH^high^ subpopulations and non-ALDH^high^ subpopulations derived from PC3M-1E8 cells and primary prostate cancer cells. (D) Western blot analysis of ALDH^high^ cells derived from PC3M-1E8 cells treated with 10 μM Stattic for 4 h. (E) Stattic significantly decreased the proportion of ALDH^high^ prostate cancer cells *in vitro*. (F) Western blot analysis of PC3M-1E8 cells treated with 10 μM Stattic for 4 h. (G) Number of ALDH^high^ cells formed from non-ALDH^high^ cells obtained by sorting PC3M-1E8 cells and primary prostate cancer cells upon treatment with IL-6 in the presence or absence of Stattic. (H) Colony formation ability of STAT3 knock-down PC3M-1E8 cells treated with 25 ng/ml IL-6. Quantitation of colonies that were at least 20 μm in diameter was recorded. *P < 0.05, **P<0.01.

To test the effect of blocking STAT3 activation on tumorigenicity of prostate cancer cells *in vitro*, a soft agar colony formation assay was performed in PC3M-1E8 cells treated with Stattic. The result showed that the inhibitory effect of Stattic on the colony formation of PC3M-1E8 was significant even at a low dose. The average number of colonies (colony was defined as >20 μm) in the 2.5 and 10 μM Stattic-treated cells was decreased by 23 and 93%, respectively (Fig. 3A).

**Table 1:** Effect of Stattic on apoptosis and cell cycle analysis in PC3M-1E8 cells

Group (n=3)	Apoptic cells, % (mean±SD)	G_1_, % (mean±SD)	S, % (mean±SD)	G_2_, % (mean±SD)
DMSO	4±0.8	44.8±5.01	38.5±4.36	16.1±3.09
2.5 μM Stattic	5.6±1.1	29±3.82	55.2±5.9^1^	13.7±3.46
5.0 μM Stattic	5.7±1.19	27.9±4.43	55.9±5.05^1^	14.6±3.01
10 μM Stattic	45.9±4.92^2^	37.5±4.3	50.2±5.38^1^	12.5±3.03

1 *P*< 0.05 versus DMSO.

2 *P*< 0.01 versus DMSO.

### Blocking STAT3 activation decreases the proportion of ALDH^high^ cells

The presence and size of the population with high ALDH enzymatic activity in PC3M-1E8 cells and clinical specimens of primary human prostate cancer was assessed by ALDEFLUOR assay. Flow cytometry demonstrated that 7.0±0.7% of PC3M-1E8 cells expressed high ALDH activity (ALDH^high^) (Fig. 3B). As shown in Table 2, ALDH^high^ subpopulations could be detected in all cell preparations obtained from clinical prostate cancer samples. Percentages of ALDH^high^ cells were highly variable and differed from patient to patient (with an average size of 1.6% to 7.1% of all cancer cells). Then the STAT3 phosphorylation of ALDH^high^ subpopulations in PC3M-1E8 cells and clinical prostate cancer samples were examined. The results showed that the ALDH^high^ subpopulations in PC3M-1E8 cells and primary human prostate cancer expressed higher levels of pSTAT3 compared with that of non-ALDH^high^ subpopulations (Fig. 3C).

**Table 2:** Flow cytometric analysis of prostate cancer cell line or specimens based on ALDH enzymatic activity

prostate cancer cell line or specimens	ALDH^high^ cells
PCa019	4.4±0.8
PCa027	7.1±1.1
PCa139	5.7±1
PCa157	2.9±0.4
PCa183	1.6±0.3

NOTE: Data are presented as percentage of cells with high ALDH enzymatic activity. Means of three individual experiments ± SD are shown.

To confirm the important role of STAT3 in prostate TICs, we next assessed the effect of Stattic on ALDH^high^ cells. The results showed that Stattic inhibited the expression of pSTAT3 in the ALDH^high^ cells (Fig. 3D). The inhibition of pSTAT3 by Stattic also downregulated the expression of its downstream targets in ALDH^high^ cells, such as survivin and c-Myc (Fig. 3D). To examine whether STAT3 inhibition might eliminate the ALDH^high^ subpopulation, we treated cancer cells with different doses of Stattic. Our results showed that Stattic effectively eliminated the ALDH^high^ subpopulation in PC3M-1E8 cells and primary cell cultures from different clinical prostate cancer samples, even at a low doses (Fig. 3E), suggesting that this subpopulation of prostate TICs is sensitive to STAT3-mediated inhibition.

Oct-4, Nanog and c-Myc are the key stemness factors and required for maintaining self-renewal and pluripotency of stem cells [22, 23]. As shown in Fig. 3F, the protein levels of Oct-4 and Nanog were significantly downregulated in PC3M-1E8 cells treated with Stattic. Study proposed that differentiated cancer cells can convert to stem-like cells to maintain equilibrium [24]. Indeed, our results showed that the addition of IL-6 to non-ALDH^high^-derived cells resulted in a generation of ALDH^high^ subpopulation in PC3M-1E8 cells and primary prostate cancer cells (Fig. 3F). The ALDH^high^ subpopulation from the non-ALDH^high^ cells were truly TICs, as defined by the *tumor-forming ability* in mice (Supplementary Table S1). However, the conversion mediated by IL-6 was significantly blocked in the presence of Stattic (Fig. 3G), and the addition of IL-6 to STAT3 shRNA lentivirus infected PC3M-1E8 cells didn't significantly increased their clonogenic capacity (Fig. 3H). The results suggest that STAT3 is important for generation of TICs from non-TICs induced by IL-6.

### STAT3 activation is required for VEGF expression in PC3M-1E8 cells

**Figure 4:**
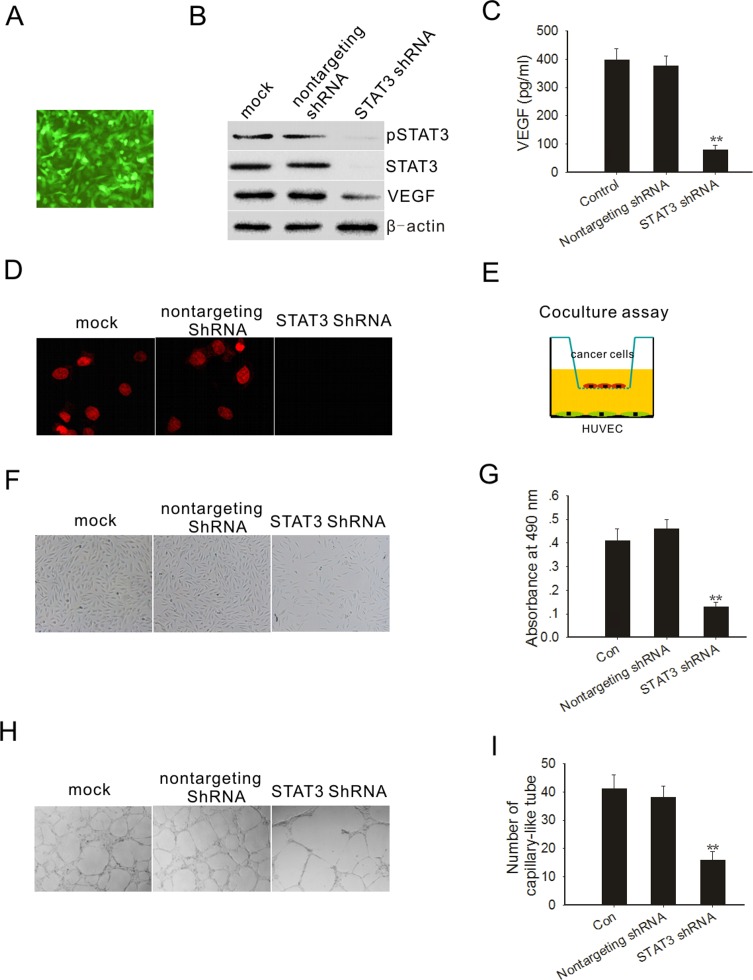
STAT3 knockdown decreased PC3M-1E8 cells mediated angiogenesis (A) PC3M-1E8 cells were transduced with a GFP lentivirus and examined by fluorescence microscopy 72 hours later. (B) Western blot analysis shows that STAT3, pSTAT3 and VEGF were downregulated in PC3M-1E8 cells transduced with STAT3 shRNA. (C) VEGF analysis by ELISA. (D) Immunofluorescence staining of pSTAT3 (red) on PC3M-1E8 cells transduced with STAT3 shRNA (E) Representative diagram of the coculture assay. (F) Representative images of cocultured HUVECs. (G) HUVECs proliferation was measured through MTT assay. (H and I) The effects of conditioned medium from PC3M-1E8 cells transduced with STAT3 shRNA on angiogenesis *in vitro*. Representative images are displayed. *P < 0.05, **P<0.01.

Angiogenesis is critical to tumor formation and maintenance [25]. We first determined whether STAT3 was required for VEGF expression in PC3M-1E8 cells. We knocked down STAT3 by RNA interference using a dicistronic lentivirus shRNA delivery system as previously described [26]. After exposure of PC3M-1E8 cells to the lentivirus encoding shRNA of STAT3 and GFP, most of the cells expressed GFP 72 hours after the infection (Fig. 4A). Cell sorting was carried out by selecting cells expressing the GFP marker at 72 hours postinfection. As shown in Fig. 4B, STAT3 and pSTAT3 protein expression were virtually depleted from the PC3M-1E8 cells after STAT3 shRNA transduction and its target protein VEGF was significantly reduced (Fig. 4B and C). In contrast, STAT3 and pSTAT3 expression were not affected by the nontargeting shRNA lentivirus (Fig. 4B). Immunofluorescence also showed that STAT3 shRNA lentivirus infected cells did not show pSTAT3 in the nucleus (Fig. 4D).

We next examined whether the knockdown of STAT3 in PC3M-1E8 cells could significantly impact endothelial cell growth. We performed a coculture experiment as previously described [27], in which PC3M-1E8 cells were cultured in an upper chamber while HUVECs were planted in the lower wells (Fig. 4E). These two chambers were separated by a permeable membrane with 0.4 μm pores, which prevented physical contact between cancer cells and endothelial cells, but allowed transfer of secreted factors. The results showed that STAT3 knockdown PC3M-1E8 cells significantly inhibit endothelial cell proliferation in comparison with control PC3M-1E8 cells (Fig. 4F and G). Endothelial tube formation assays showed that the pro-angiogenic potency of STAT3 knockdown PC3M-1E8 cells was significantly inhibited as compared with control PC3M-1E8 cells (Fig. 4H and I).

### STAT3 knockdown impairs prostate tumor cell tumorigenicity *in vivo*

**Figure 5:**
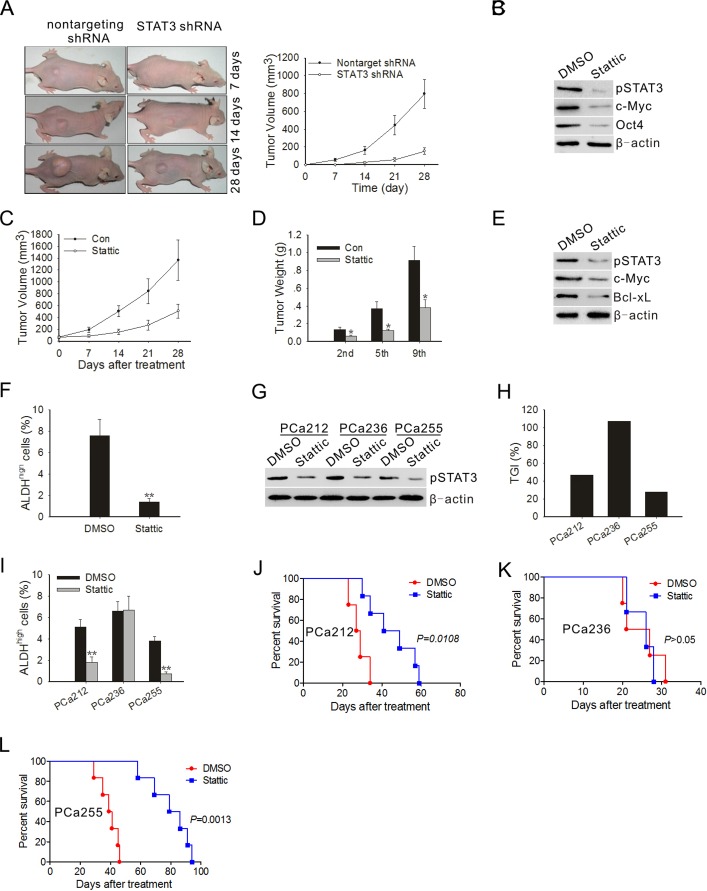
STAT3 blockade inhibited the development of tumor and ALDH^high^ subpopulation cells *in vivo* (A) Representative images of prostate tumor from nude mice that received subcutaneous injections of PC3M-1E8 cells infected with STAT3 shRNA-expressing lentivirus or control lentivirus (left panel). Tumor volumes were measured after tumor cell inoculation every 7 days for a period of 4 weeks (right panel). Error bar represents SD (n = 5). (B) Immunoblots of pSTAT3, c-Myc and Oct4 in xenograft tumors at the end of the experiment. (C) Measurement of subcutaneous xenograft tumor size after treatment with Stattic. Error bar represents SD (n = 5). (D) Tumor bearing mice (n=3) were sacrificed after 2nd, 5th and 9th injection to assess tumor growth. (E) Immunoblots of pSTAT3, c-Myc and Bcl-xL in xenograft tumors treated with DMSO or Stattic on day 28 following first treatment. Representative data from 1 of 3 independent experiments are shown. (F) Xenograft tumors (n=3) were subjected to enzymatic dissociation, ALDEFLUOR staining and flow cytometry assay on day 28 following first treatment. (G) Immunoblots of pSTAT3 in xenograft tumors treated with DMSO or Stattic on day 28 following first treatment. Each lane represents the tumor lysate of an individual experimental mouse. Representative data from 1 of 3 independent experiments are shown. (H) TGI of Stattic in the F3 generation of three patient tumors. Five mice were used in each experimental group. (I) Patient-derived xenograft tumors (n=5) were subjected to enzymatic dissociation, ALDEFLUOR staining and flow cytometry assay on day 28 following first treatment. (J~L) Kaplan-Meier survival curves of mice (n = 8 for each group) implanted with human primary prostate tumors after Stattic treatment. The log-rank test was used to determine differences between Kaplan-Meier curves. *P < 0.05, **P<0.01.

To investigate the effect of STAT3 knockdown on prostate tumor cell tumorigenicity, we injected either STAT3 knock-down or control PC3M-1E8 cells subcutaneously into athymic nude mice and monitored tumor growth over 4 weeks. As shown in Fig. 5A, PC3M-1E8 cells transduced with nontargeting shRNA control rapidly formed tumor, in contrast, PC3M-1E8 cells transduced with STAT3 shRNA displayed increased tumor latency and decreased tumor growth rate, concomitant with reduction of CSC marker expression in tumor (Fig. 5B).

### Blocking STAT3 signaling suppresses tumor growth and inhibits development of ALDH^high^ subpopulation in both cell-line xenograft models and PDTX models

To determine if the effects of blocking STAT3 activation on tumor cells were applicable to tumors *in vivo*, we first tested tumor xenografts in athymic nude mice inoculated with PC3M-1E8 cells. Compared with control tumors, those in mice, having received Stattic, displayed strong growth inhibition (Fig. 5C). For accurate measurement of tumor growth, mice of control and Stattic treated groups were sacrificed to determine tumor weight after receiving the second, fifth or ninth treatment. As shown in Fig. 5D, when tumors in the control group expanded and those in Stattic treated group were significantly arrested. Control animals developed symptoms such as anorexia and weight loss before they were put down, whereas Stattic-treated animals remained symptom free. Confirming the *in vitro* findings, western blotting of tumor lysates also revealed a significant reduction in pSTAT3 protein levels and its downstream target proteins in mice treated with Stattic (Fig. 5E). We used flow cytometry to determine the percentage of ALDH^high^ subpopulation in the tumors treated with vehicle or Stattic. The results showed Stattic treatment significantly reduced the percentage of ALDH^high^ cells (Fig. 5F).

Next, we further analyzed the effect of Stattic on tumor growth in PDTX models. The ALDH^high^ subpopulations in three patient-derived xenografts were detectable to various extents (Supplementary Table S2). However, within a given patient xenograft lineage, the relative percentage of ALDH^high^ subpopulation remained conserved through F1 to F3 passages in mice (Supplementary Table S2), suggesting that the xeno-trans-plantation process did not affect ALDH expression. Western blotting of tumor lysates showed that high pSTAT3 protein levels were found in all patient-derived F3 xenografts (Fig. 5G). To determine the extent of pSTAT3 inhibition by Stattic in the three individual patient-derived tumors, Western blot analysis of pSTAT3 in xenograft tumors was performed at the end of the experiments. As shown in Fig. 5H, *in vivo* treatment with Stattic greatly decreased the levels of pSTAT3 protein in the three individual patient-derived tumors. Treatment with Stattic resulted in a significant decrease in tumor volume in PCa212 and PCa255 xenografts, the TGI varied from 47% to 28% (Fig. 5H), and the percentage of ALDH^high^ subpopulation in the two tumors was also reduced significantly (Fig. 5I). Kaplan-Meier curves showed the increase in survival of mice bearing PCa212 and PCa255 xenografts treated with Stattic (Fig. 5J-L).

## DISCUSSION

Presently, there are no effective pharmacological therapies for metastatic and castration-resistant prostate cancer [28]. Therefore, it is important to explore the molecular mechanisms involved in the pathogenesis and progression of this disease to identify novel therapeutic targets and develop effective treatment strategies.

In this study, we first investigated whether constitutive activation of STAT3 protein is associated with prostate cancer *in vivo*. Our findings are consistent with the study showing significantly higher levels of constitutively active STAT3 in primary prostate tumor specimens compared to matched adjacent nontumor tissues [29]. Using the STAT3 small molecule inhibitor, Stattic, which has been shown to selectively inhibit the function of the STAT3 SH2 domain regardless of STAT3 phosphorylation status [30], we showed that Stattic potentially prevented phosphorylation of STAT3 in prostate cancer cells. The Tyr705 phosphorylation of STAT3 results in its homodimerization. Then the STAT3 dimer translocates to the nucleus for binding to specific DNA sequences on the promoter of its target genes [9, 10]. Our study indicated that the Stattic-mediated inhibition of STAT3 phosphorylation suppressed its transcriptional activity as evidenced by the decrease in the constitutive nuclear translocation of pSTAT3 and the suppression of the STAT3-regulated gene products (e.g., Bcl-xL, survivin and c-Myc) in Stattic-treated PC3M-1E8 cells. In addition, STAT3 shRNA was used to inhibit the STAT3 expression and activity. Our results showed that STAT3 shRNA also inhibited STAT3 phosphorylation and STAT3 downstream genes expression in PC3M-1E8 cells. Levels of IL-6 are elevated in the serum of patients with hormone-refractory and metastatic prostate carcinoma [31, 32]. In the present study, it was found that IL-6 was able to induce STAT3 activation, but the activation could be blocked by Stattic in a dose-dependent manner in PC3M-1E8 cells.

In agreement with the inhibition of STAT3 activation and nuclear localization, Stattic caused S-phase accumulation at low-dose levels and led to massive apoptosis at a relatively high-dose level in PC3M-1E8 cells. In view of toxicity of anticancer drugs, optimal scheduling is potentially useful in improving these treatments. One of the conceivable strategies of protocol optimization, exploiting drug specificity, is to arrest cancer cells in the S phase [33]. As shown in the present study, Stattic may cause S-phase arrest in PC3M-1E8cells, thus, it may be a useful therapeutic tool in human prostate cancer. In addition, we also demonstrated that the proapoptotic response to Stattic in PC3M-1E8 cells was not significantly impaired by IL-6–mediated activation of STAT3.

While the successful cancer cures require eliminating all tumor cells, TICs may represent particular therapeutic challenges. Optimal development of specific agents against TICs may involve targeting critical regulators or signaling pathways for the maintenance of stem cells. For prostate cancer, the main effort to target constitutive STAT3 signaling is only focused on the bulk of cancer cells at the present time. Our study demonstrated that human prostate cancer cell line and surgical specimens contained a subpopulation of ALDH^high^ cells which expressed higher level of pSTAT3. The effective elimination of the subproportion of ALDH^high^ cells in the PC3M-1E8 cells and primary prostate cancer cells by Stattic suggested that these TIC-like cells were sensitive to STAT3 inhibition. Recently, researchers proposed that differentiated cancer cells (non-TICs) are able to convert to stem-like cells to maintain equilibrium [24, 34]. These results imply that removing TICs may prompt non-TICs in the tumor to convert into stem cells to maintain the equilibrium [24]. We found that the expression of stem cell markers was effectively suppressed by Stattic and STAT3 played an important role in IL-6-mediated conversion of non-TICs to TICs. Therefore, we suggested that targeting STAT3 therapy not only directly killed existing TICs, but that it indirectly lowered the TIC burden by inhibiting the conversion of non-TICs to TICs.

The perivascular niche plays a vital role in controlling the unique properties of TIC populations [35]. In this study, we demonstrated that STAT3 contributed to the ability of prostate cancer to support angiogenesis. Thus, systemic inhibition of STAT3 may not only directly target prostate CSC self-renewal, but may also disrupt the functional vascular niches that are essential for prostate CSC maintenance. It is unlikely that anti-CSC therapies will be effective in isolation, therefore, the potential effects of STAT3 inhibition on angiogenesis may offer synergies.

To test the tumor dependence on STAT3, we used shRNA to specifically knock down STAT3. Our results showed that the blockade of STAT3 significantly impaired the ability of prostate cancer cells to initiate development of prostate adenocarcinoma. PDTX tumours maintain the molecular, genetic and histological heterogeneity typical of tumours of origin through serial passaging in mice [36]. The tumour histology of PDTX models provides an excellent *in vivo* preclinical platform to study CSC biology and novel cancer therapeutics [36]. Using the PDTX model, we presented the results with three patient-derived carcinomas treated with Stattic, simulating the first stage of a classic human two-stage phase II clinical trial. The data from our study showed that Stattic was capable of suppressing tumor growth, increasing survival and reducing the percentage of ALDH^high^ subpopulation in PDTX models. These results indicate that STAT3 is required for maintenance of the tumorigenic phenotype.

Our findings strongly suggest that targeting STAT3 signals may be useful as both TICs and non-TICs directed therapy. Our studies provide evidence that inhibiting STAT3 pathways should be considered for further exploitation in therapeutic development of prostate cancer.

## MATERIALS AND METHODS

### Clinical tissues

Human primary prostate tumors and adjacent nontumor tissues were obtained after obtaining informed consent according to protocols approved by the Ethics Committee of Tongji Hospital, Tongji Medical College. The fresh tumor specimen were collected, either at the time of surgical resection or by a tumor biopsy and prepared for implantion in immunodeficient mice, single-cell suspension as described [8, 37] or immediately frozen in liquid nitrogen and stored at –80°C. All specimens were examined by a pathologist and confirmed the malignant and non-malignant tissues. None of these patients had received preoperative chemotherapy or preoperative radiation therapy.

Because of the priority assigned to the pathological evaluation, for each patient only a limited amount of tissue was available for investigation. Therefore, not all of the assays listed below could be performed in the same patients.

### Cell culture

PC3M-1E8 prostate cancer cells were obtained from the China Center for Type Culture Collection (Shanghai, People's Republic of China) and A2780 ovarian cancer cells were purchased from American Type Culture Collection (Rockville, MD, USA). Cells were cultivated according to the supplier's instructions. Human umbilical vein endothelial cells (HUVECs) were isolated as previously described [38]. All cells were maintained in a 37°C atmosphere containing 5% CO_2_. Cells were used at low passages and not cultured for more than two months. Cells were routinely tested for mycoplasma and found free of mycoplasma. Stattic, a previously reported STAT3 inhibitor [30], was purchased from Sigma (Sigma Chemical Co., St. Louis, MO).

### Lentiviral infections

Lentiviruses were prepared and infected PC3M-1E8 cell as described [26]. The plasmids pCMVD8.2 (GAG-POL DNA), the vesicular stomatitis virus (VSV-G) envelope plasmid pMD.G and gene transfer plasmid pLVTH were provided by Dr. Ralph B. Arlinghaus at the University of Texas. The STAT3 shRNA sequence and the nontargeting sequence used in this study were synthesized, according to the published sequence [39]. The PC3M-1E8 cells were infected with lentiviruses in the presence of 8 μg/ml of polybrene (Sigma Chemical Co., St. Louis, MO). After 72 hours of infection, PC3M-1E8 cells were selected by fluorescence-activated cell sorting using GFP as the marker.

### Western blot analysis

Standard western blot analysis was performed with antibodies for STAT3, pSTAT3 (Tyr705), vascular endothelial growth factor (VEGF), survivin, c-Myc, Nanog, Oct-4, β-actin (Santa Cruz, CA) and Bcl-xL (Cell Signaling Technology, Danvers, MA). The protein bands were detected by enhanced chemiluminescence (Pierce Biotechnology, Rockford, IL).

### Apoptosis and cell cycle analysis

Fluorescein isothiocyanate-annexin V and propidium iodide staining (BD Biosciences Pharmingen, San Diego, CA) were used to determine apoptosis and to analyze the DNA content.

### Immunoluorescence staining

Immunoluorescence staining was preformed as previously described [40].

### Colony formation assay

Colony formation assay was performed as previously described [1]. The plates were fed weekly with 0.5 ml of DMEM/10% FBS containing the indicated concentrations of Stattic.

### ALDEFLUOR assay

For ALDH staining, cells were trypsinized to single cells and subsequently suspended in ALDEFLUOR assay buffer containing ALDH substrate and then incubated for 35 minutes at 37°C, following the manufacturer's instructions (StemCell Technologies, Aldagen, Inc., Durham, NC). After staining, cells were kept in ice during all subsequent procedures. For each experiment, a sample of cells was stained under identical conditions with a specific ALDH inhibitor, diethylaminobenzaldehyde (DEAB), as a ALDH-negative control, following the manufacturer's instructions. The amount of intracellular fluorescence was measured by flow cytometry. For ALDEFLUOR assay in xenograft tumors by flow cytometry, in order to eliminate cells of mouse origin from the xenograft tumors, staining with an anti-H2Kd antibody was used followed by staining with a secondary antibody labeled with PE, both from BD Pharmingen.

### *In vitro* angiogenesis evaluation

*In vitro* formation of tubular structures was studied on an extracellular matrix as described below. Briefly, 24-well plates were coated with cold matrigel (BD Biosciences) (80 μl/well of a solution of 9:1 Matrigel to 10 × buffer). Then, 1 × 10^5^ HUVECs, suspended in the conditioned media collected from cell cultures of PC3M-1E8 cells transduced with STAT3 shRNA, were seeded in each well. After 16 hours incubation, angiogenesis was assessed based on formation of capillary-like structures. The number of capillary-like tubes was counted in five randomly chosen microscopic fields (Nikon Eclipse, TE2000-U), and the average was calculated.

### Xenograft tumor models

The animal studies were approved by the Ethics Committee of Tongji Hospital, Tongji Medical College. Athymic BALB/c nude mice were purchased from the Animal Experimental Center of Slaccas (Shanghai, People's Republic of China). The 4- to 6-week-old male mice were maintained in laminar flow cabinets under specific pathogen free conditions. For tumorigenicity study, after lentiviral infection and selection, 4 × 10^6^ PC3M-1E8 cells were subcutaneously injected into mice in 100 mL of BD matrigel. Tumor growth was monitored weekly for 4 weeks. To detect the effects of STAT3 inhibition *in vivo*, 4 × 10^6^ PC3M-1E8 cells were inoculated into mice in an identical fashion as described above and allowed to develop into a tumor of 5–6 mm in diameter before treatment. Then, mice were randomly distributed into two treatment groups: (a) control vehicle (100% DMSO) and (b) 20 mg/kg of Stattic. Stattic or DMSO was administered via intraperitoneal injection on a Q3D × 9 schedule. The tumors were measured in two dimensions with calipers weekly, and the volume was calculated as length × width^2^ × 1/2. The PDTX models were established as previously described [37]. Xenografts from the third generation (F3) were measured with ultrasound and allowed to grow to a size of ~200 mm^3^, at which time the animal models were treated in an identical fashion as described in PC3M-1E8 xenograft model studies. Five mice was euthanized and the tumors were harvested on day 28 following first treatment. Relative tumor growth inhibition (TGI) was calculated by relative tumor growth of treated mice divided by relative tumor growth of control mice (T/C). Eight mice per group were monitored for death and were euthanized when they appeared moribund.

### Statistical analysis

All values were given as means ± SD of at least three independent experiments. The significance of differences between groups was assessed by two-tailed Student's *t*-test. Statistical significance was defined as *p* < 0.05. Survival was analyzed by the Kaplan–Meier method and differences were analyzed by a log-rank test.

## SUPPLEMENTARY MATERIAL AND TABLES


