# Supplementary material for: Inhibition of STAT3 signaling targets both tumor-initiating and differentiated cell populations in prostate cancer
Source: Oncotarget. 2014 Aug 6;5(18):8416–28. doi: 10.18632/oncotarget.2314 (PMC4226693; doi:10.18632/oncotarget.2314)
Supplement: Supplementary file 1 [file oncotarget-05-8416-s001.pdf]

Inhibition of STAT3 signaling targets both tumor-initiating and differentiated cell populations in prostate cancer

Supplementary Material

Table S1: Tumor formation in mouse xenografts 6 weeks after injection of the indicated number of sorted non-ALDH^{high} cells and and IL6-derived ALDH^{high} cells derived from PC3M-1E8 prostate cell line and primary prostate cancer cells.

cell popolation	10^3 Tumors	10^4 Tumors
non-ALDH^{hig} (PC3M-1E8)	0/6	1/6
ALDH^{high} (IL-6) (PC3M-1E8)	2/6	5/6
non-ALDH^{hig} (PCa027)	0/6	0/6
ALDH^{high} (IL-6) (PCa027)	3/6	6/6

Table S2: Analysis of ALDH activity in different generations of patient-derived xenograft tumors.

Patient	Xenograft generation	ALDH expression
PCa212	F1	5.3±0.9
	F1	4.6±0.5
	F2	5.1±0.7
	F2	4.8±0.5
	F2	5.6±1.0
	F2	4.3±0.7
	F2	5.2±0.6
	F3	4.7±0.5
	F3	5.5±0.7
	F3	6.1±0.9
	F3	4.1±0.6
	F3	4.9±0.8
	F3	5.0±0.9
PCa236	F1	6.1±1.2
	F2	5.2±0.7
	F2	7.0±1.7
	F2	6.3±0.9
	F2	6.9±1.0
	F2	5.4±0.9
	F3	7.0±0.8
	F3	5.9±0.7
	F3	7.5±1.6
	F3	5.1±1
	F3	6.8±1.1

	F3	6.0±0.9
PCa255	F1	3.5±0.5
	F2	3.7±0.6
	F2	3.1±0.4
	F2	2.9±0.5
	F2	3.9±0.7
	F3	4.0±0.8
	F3	3.5±0.9
	F3	2.8±0.6
	F3	3.2±0.5
	F3	4.4±0.9

NOTE: Data are presented as percentage of cells with high ALDH enzymatic activity. Means of three individual experiments ± SD are shown.

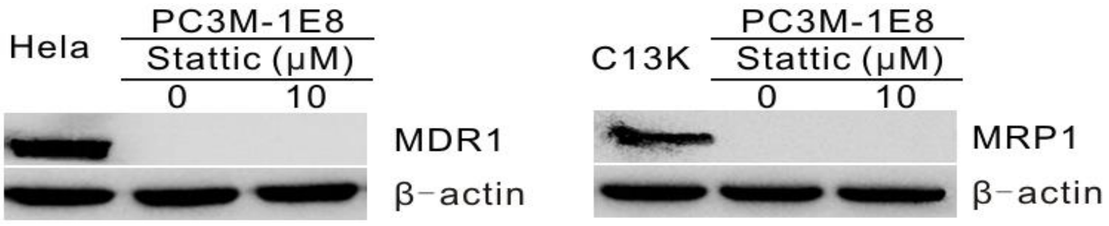

Supplementary Figure1:
